# Results of an adapted whole-body MRI in the primary care of children and adolescents in the trauma room

**DOI:** 10.1186/s13018-025-05802-3

**Published:** 2025-04-19

**Authors:** Silvia J. Hufnagel, Alexis Brinkemper, Simon Pätzholz, Charlotte Cibura, Volkmar Nicolas, Thomas A. Schildhauer, Christiane Kruppa

**Affiliations:** 1 Department of General and Trauma Surgery, BG University Hospital Bergmannsheil Bochum, Ruhr-University Bochum, Bürkle de la Camp-Platz 1, 44789 Bochum, Germany; 2 Department of Radiology and Interventional Radiology, BG University Hospital Bergmannsheil Bochum, Ruhr-University Bochum, Bürkle de la Camp-Platz 1, 44789 Bochum, Germany

**Keywords:** Polytrauma, Children and adolescents, MRI, Trauma room

## Abstract

**Background:**

In diagnostic imaging as part of pediatric polytrauma management radiation exposure and diagnostic benefit of a whole-body computed tomography must be weighed up against each other. Performing an adapted polytrauma magnetic resonance imaging (MRI) could be a sensible alternative in some cases. The aim of this study was to show what findings are made in the MRI and if this has consequences for further diagnosis and therapy.

**Methods:**

We performed a retrospective evaluation of the adapted polytrauma MRI examination as part of the primary survey in trauma room care of children and adolescents (indication was made individually) between 05/2016 and 12/2022 in a level 1 trauma center. Demographic data, cause of the accident, findings obtained during the MRI, additive radiological diagnostics performed, time between admission to hospital and MRI and the therapeutic consequence of the MRI findings were evaluated.

**Results:**

33 children (21 boys, 12 girls) with an average age of 11.3 years (2.9–17.6 years) were evaluated. The majority of accident mechanisms were traffic accidents in 14 (42.4%) cases and a fall from a height of > 3 m in 6 cases (18.2%). Additional radiological diagnosis was performed in 20 (60.6%) cases. Time between admission and MRI was in average 47.58 min. In 23 (69.7%) patients, 31 injuries were detected on MRI such as spinal injury (9 cases), soft tissue injury (9), skull/brain injury (4), bony lesion of the extremities/shoulder girdle (4), pelvic injury (3), and lung injury (2). The additional injuries identified on MRI did not lead to surgical intervention in any case.

**Conclusions:**

It seems reasonable to perform an MRI in children admitted to the trauma room, if available and if the children are circulatory stable. In particular, injuries of the spine and pelvis can be detected without additional radiation diagnostics, even if they usually do not require surgical intervention.

**Trial registration:**

DRKS, DRKS00036020. Registered 28 January 2025—Retrospectively registered, https://www.drks.de/DRKS00036020.

## Background

Imaging in children (0 to 17 years) with trauma, which is performed in the trauma room as part of polytrauma management, gives rise to discussion. Although all relevant injuries are usually detected by computed tomography (CT), an indication for surgery is rarely made from the CT findings [1, 2]. Even clinically significant injury is not found in the majority of patients [3, 4]. The radiation-exposing performance of a whole-body CT is therefore criticized [2, 3]. Due to the higher radiation sensitivity in children, radiation exposure should be as low as reasonably achievable (ALARA-principle) [5, 6]. An increased risk for leukemia and brain tumors after CT scans during childhood is well described in literature [7–9]. However, since children are often more difficult to assess after trauma, CT examinations are sometimes performed more frequently than in adults [2]. That the difficulty of clinical assessment of injury severity in children plays a role is also shown by another study in which it was demonstrated that a whole-body CT is performed more frequently in adult trauma centers than in pediatric trauma centers - with no difference in patient outcomes [10].

CT protocols are already being adapted for children [11, 12]. Meanwhile, research is still divided on whether selective scanning is advantageous over whole-body CT even in terms of mortality [3, 13, 14]. Especially in children and adolescents with stable circulation and no obvious injuries in the trauma room, there is often the question of which imaging is required. First sonography (extended Focused Assessment with Sonography for Trauma (eFAST)) as standard diagnostics is performed, but often uncertainty about injuries remains after this. X-ray images can be taken afterwards. However, especially in the case of pain in the spine or pelvis, X-rays cannot rule out injuries in this area with certainty. Therefore sectional imaging is needed. In the past, a CT scan with corresponding radiation exposure was usually performed [15, 16]. This can be eliminated by performing an adapted polytrauma MRI instead. However, the limited availability and delay of therapy are raised against this [17].

The aim of this study is to identify what findings an adapted polytrauma MRI yield, if available and feasible. Are there differences regarding age groups? Besides, we wanted to know if there are age-related differences and whether the findings have therapeutic consequences.

## Methods

The present study was performed in accordance with the Declaration of Helsinki. Ethical permission for this study was obtained from the local ethics committee. We performed a retrospective evaluation of an adapted polytrauma MRI examination. It was part of the primary survey in trauma room care of children and adolescents in a level 1 trauma center. All patients aged 0 to 17 years in the period between 05/2016 and 12/2022 by whom an MRI in the trauma room was performed as part of the primary survey were included. The decision to indicate a whole-body MRI was made individually in the trauma room by the team on duty. Only children and adolescents with stable circulation who did not obviously require immediate surgical intervention were selected. Not all of these patients would have received a whole-body CT instead of the MRI scan. In addition to the demographic data and cause of the accident, the findings obtained during the MRI were evaluated. The MRI was analyzed by an advanced radiologist (senior physician). Furthermore, the additive radiological diagnostics performed pre- and post-MRI, the time between admission to hospital and MRI and the therapeutic consequence of the MRI findings were evaluated.

The MRI device used was a Siemens Aera 1.5 Tesla MRI. A head/neck coil, an integrated spine coil and an additional flexible body coil were used. A standard protocol consisting of the following sequences was applied:

- head: coronal and axial t2 sequences, axial t1 sequence, diffusion weighted sequences.
- whole-body: coronal fat suppressed t2 sequences head/neck, thorax and abdomen, sagittal t2 sequences Cervical, thoracic and lumbar spine with and without fat suppression.

Using the standard protocol each MRI took approximately 21:15 min.

Statistical analyses were performed using PSPP 1.6.2 (GNU Project, Free Software Foundation, Inc.) and SPSS Statistics 30.0.0.0 (IBM Corp., Armonk, NY: IBM Corp). Data are given as mean ± standard deviation (SD). The age is presented as mean, range and median. All continuous variables were normally distributed, as demonstrated by the Kolmogorov-Smirnov test. A t-test or one-way ANOVA with Bonferroni correction for multiple group comparison was performed. Evaluation of categorical variables was carried out using the Fisher’s exact test. Results were considered statistically significant when the p value was < 0.05.

## Results

33 children (21 boys, 12 girls) with an average age of 11.3 years (2.9–17.6 years, median: 11.9 years) were evaluated who received an adapted polytrauma MRI as part of the trauma room management. The main part of the trauma mechanisms was a traffic accident in 14 (42.4%) cases and a fall from a height of > 3 m in 6 cases (18.2%). Other trauma mechanisms were a fall from a height of < 3 m and sports accidents (soccer, trampoline, wrestling). Patients’ demographics and trauma mechanisms are shown in Table 1.

**Table 1:** Demographics and trauma mechanism

Characteristic	Mean (SD; range) or *n* (%)
Female	12 (36.4%)
Male	21 (63.6%)
Age (years)	11.3 (4.1; 2.9–17.6)
Age group	
0–6 years (1)	5 (15.2%)
7–12 years (2)	14 (42.4%)
13–17 years (3)	14 (42.4%)
Trauma mechanism	
Traffic accident	14 (42.4%)
Fall from a height of > 3 m	6 (18.2%)
Fall from a height of < 3 m	4 (12.1%)
Sports accident	3 (9.1%)
Other trauma mechanism/ unclear	6 (18.2%)

Additional radiological diagnostics were performed in 20 (60.6%) patients. Of these, 12 patients (36.4%) underwent only an X-ray examination, 4 got an additional CT (12.1%) and 4 patients needed both - additional X-ray and CT (12.1%). In 15 patients it was performed after MRI (11 (33.3%) cases X-ray, 4 (12.1%) cases CT). In most cases (4), the CT examination prior to the MRI was a CCT to quickly rule out head injuries. A CT scan after the MRI was usually performed in response to a finding from the MRI.

Average time from the admission of the child to the hospital until the MRI was performed was 47.58 min (± 24.36 min). Hereby 16 patients were admitted to hospital during regular working hours between 8 a.m. and 4 p.m. and 17 patients were admitted during on duty hours. There were no significant differences between those two time slots (between 8 a.m. and 4 p.m.: 48.75 min (± 27.65 min), during on duty hours: 46.47 min (± 21.62 min); *p* = 0.793). Regarding the age groups mentioned above, the youngest patients (0–6 years) showed the longest time between admission and MRI (55.80 min). However, this was not significant in comparison with the other age groups (49.07 min and 43.14 min, *p* = 0.596). Mean times and p values are presented in Table 2.

**Table 2:** Mean time between admission to hospital and MRI by age groups

Age group	Mean time in min (± SD)	*P* value ANOVA	*P* value Bonferroni Correction
0–6 years (1)	55.80 (± 12.03)	0.596	1–2: 1.0
7–12 years (2)	49.07 (± 23.66)		1–3:1.0
13–17 years (3)	43.14 (± 28.40)		2–3: 1.0

In 23 (69.7%) patients, an injury was detected on MRI. 7 patients suffered more than one injury. Looking at the age groups, injuries were detected more frequently in the 0–6 age group than in the 7–12 age group (see Table 3). In group 1 there was an injury detected in all cases whereas in group 2 it was only detected in 50% of cases. Age group 3 showed an injury in 78.6% of the analyzed cases. No increased frequencies compared to the expected values were found according to Fisher’s exact test.

**Table 3:** Injury detected by age groups

Age group	Injury detected	No injury detected	Fisher’s exact test
0–6 years (1)	5	0	0.081
7–12 years (2)	7	7	
13–17 years (3)	11	3	

**Fig. 1:**
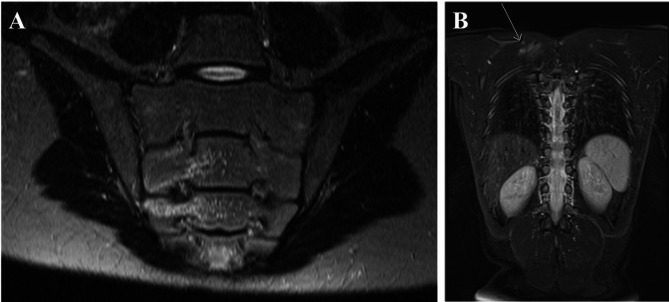
Examples of injuries found in the whole-body MRI after polytrauma

Following types of injury have been detected: A soft tissue injury was present in 9 cases, such as hematomas or contusions. Lung injury was diagnosed 2 times and a bony lesion of the extremities/shoulder girdle 4 times. A pelvic injury could be demonstrated in 3 patients and spinal injury in 9 times. In 5 patients, a spinal cord lesion could be excluded in the presence of neurologic deficits. Intraabdominal free fluid without parenchymal injury was detected 3 times and skull/brain injury was present 4 times. Number and type of injuries are shown in Table 4. The additional injuries identified on the MRI did not lead to surgical intervention in any case and treatment remained conservative. Figure 1 shows two examples for findings in the MRI.

**Table 4:** Number and type of injuries detected by MRI

Type of injury	Patients *n* (%)
Soft tissue injury	9 (27.3%)
Lung injury	2 (6.1%)
Bony lesion of the extremities/shoulder girdle	4 (12.1%)
Skull/brain injury	4 (12.1%)
Pelvic injury	3 (9.1%)
Spinal injury	9 (27.3%)

A: 16-year-old boy, motorcycle accident. In the whole-body native MRI evidence of impaction in the area of the right massa lateralis of the sacral bone. Muscle hematoma (T2 sequence). B: 8 year old boy, hit as a pedestrian by motorcycle. In the whole-body native MRI evidence of a muscle hematoma in the area of the shoulder girdle (T2 sequence with fat suppression). No further evidence of trauma sequelae.

## Discussion

When a child suffers a trauma, no injury should be missed. But on the other hand radiation exposure especially in children should be as low as possible [5, 6]. This means a challenge in case of a pediatric polytrauma. The management of diagnostic imaging in the trauma room is not always easy. A whole-body CT is often done to be on the safe side and because it is the standard procedure for adults [15, 16]. A recent study shows an average effective dose of 18.14 ± 10.44 mSv [18] for a whole-body CT in pediatric trauma patients, whereby the effective dose in the group of 1–5 years was significantly higher than in the group from 15 to 18 years [18]. Former studies show a mean for the total effective dose per patient of 14.9 mSv [19]. Komut et al. also show that in 72.4% of the patients no pathologies of clinical importance were determined [18]. Therefore, the whole-body CT is repeatedly criticized and the question is whether and when this is necessary. Therefore a whole-body MRI could be an alternative method. In this regard, we were able to show in our study that many injuries could be found on an adapted polytrauma MRI and subsequently diagnosed and treated accordingly.

After individual indication an adapted polytrauma MRI was performed as part of the primary survey in our hospital. In 20 cases (60.6%) additional radiographic diagnostic was performed. Overall, this is a high rate of cases, but since primarily individual regions were visualized by X-ray (36.4% only X-ray) or a specific region by CT (12.1% only CT, 12.1% additional CT and X-ray), the radiation exposure is still significantly reduced. This is in line with the findings of Ludwig et al., who found that 54% of cases received additional radiographs (45% X-ray, 11% CT) [7].

A recent study shows that a whole-body MRI took 2.5 times longer from patient`s arrival until scan than a whole-body CT (92.1 vs. 37.1 min) [17]. Another study found a mean time between arrival in the emergency department an MRI of 71 min (± 132 min) [7]. We could show a reasonable lower time (47.58 min) from admission to hospital to MRI. The reasons for this could be the comparatively low number of patients and, in particular, the small number of very young patients (15.2% age group 0–6 years). In addition, the distance in our emergency department between trauma room and MRI is very short. As level 1 trauma center a MRI is available round the clock. There was no difference regarding the time slot of arrival, although we would have expected the time to be a longer during on-duty hours. Also there were no significant differences regarding age groups. Time for the youngest patients (age group 1, 0–6 years) was longer than for the other two groups (55.80 min vs. 49.07 min/ 43.14 min), but without statistical significance.

Main causes of the accidents were traffic accidents (42.4%) and falls (> 3 m 18.2%, < 3 m 12.1%). Injury was recorded in 72.7% in our study, with a higher percentage of injuries in the group aged between 0 and 6 years compared to the older age groups. Ludwig et al. showed a comparable rate of 68% injuries on MRI in relation to the total number of children examined after high-energy trauma [7]. Another study comparing whole-body CT and whole-body MRI in pediatric polytrauma diagnostics shows that the MRI was significantly preferred at younger ages (9.6 vs. 12.8 years; *p* < 0.001) [17]. Since we were able to show that findings are also more frequent in the young age group, it seems sensible to indicate an MRI here in particular. One reason could be that very young patients are more difficult to assess.

Types of injury detected particular in our study were soft tissue injury (27.3%), spinal injury (27.3%), bony lesion of the extremities/shoulder girdle (12.1%), skull/brain injury (12.1%) and pelvic injury (9.1%). Another study shows a higher rate for cranial injuries (27%) [7]. It could be a reason that head traumas in our clinic are more likely to have a CT scan, since head injuries are the most common cause for traumatic death in pediatric patients [20]. In our study, all patients who were diagnosed by MRI were treated conservatively. Even if this did not result in a surgery, appropriate treatment could be initiated, which would not have been possible without cross-sectional imaging. This seems particularly relevant for the spinal and pelvic injuries found. MRI is even more sensitive than CT in this respect. Additional to the findings for spinal and pelvic injury mentioned above (36.4% in total), in 5 patients, a spinal cord lesion could be excluded in the presence of neurologic deficits.

Our study has limitations. The study group is rather small despite a long study period. This shows that the whole-body MRI was not a standard examination for our trauma room team. Nevertheless, it is important to conduct such a study in order to gain initial insights into its feasibility. For example, we were able to provide promising figures for the time between admission to hospital and MRI. However, due to the small total number, the subgroups investigated are of course also very small, which significantly reduces the informative value. Another limitation is the retrospective design and the fact that we did not examine a comparison group, for example with CT diagnostics. Furthermore, it should be noted that an MRI is only considered in cardiopulmonary stable children, as in addition to the longer duration of a whole-body MRI compared to a CT, the monitoring options in the MRI are also worse. This is also confirmed by another study in which it was shown that the Injury Severity Score was higher, when a whole-body CT was performed in comparison to a whole-body MRI (10.6 vs. 5.8; *p* = 0.001) - but without any significant difference in mortality [17]. Higher costs of the MRI in comparison to a CT and the additional sedation required for some children could be reasons against performing an MRI as well. Further studies with larger numbers of cases are needed to confirm our results. For the future, it would be useful to develop a kind of checklist of cases in which a whole-body MRI should be performed. We have already been able to highlight a few points in this regard.

## Conclusions

If an MRI is available and the children are circulatory stable, it seems reasonable to perform it especially in younger children. In particular, injuries of the spine and pelvis can be detected without additional radiation diagnostics, which are poorly accessible to native diagnostics, even if they usually do not require surgical intervention. However, in children and adolescents with circulatory instability, the duration of the examination remains the limiting factor.

## Data Availability

All data generated or analysed during this study are included in this published article [and its supplementary information files]. Raw data that support the findings of this study are available from the corresponding author, [CK], upon reasonable request.

## Abbreviations

MRI: magnetic resonance imaging
CT: computed tomography
ALARA: as low as reasonable achievable
eFAST: extended Focused Assessment with Sonography for Trauma
SD: standard deviation
min: minutes
a.m.: ante meridiem
p.m.: post meridiem
mSv: millisieverts
